# Visualizing a Team's Goal Chances in Soccer from Attacking Events: A Bayesian Inference Approach

**DOI:** 10.1089/big.2018.0071

**Published:** 2018-12-13

**Authors:** Gavin A. Whitaker, Ricardo Silva, Daniel Edwards

**Affiliations:** ^1^ Department of Statistical Science, University College London, London, United Kingdom.; ^2^ Stratagem Technologies, London, United Kingdom.; ^3^ The Alan Turing Institute, London, United Kingdom.

**Keywords:** Bayesian inference, Gaussian mixture model, spatial modeling, soccer

## Abstract

We consider the task of determining the number of chances a soccer team creates, along with the composite nature of each chance—the players involved and the locations on the pitch of the assist and the chance. We infer this information using data consisting solely of attacking events, which the authors believe to be the first approach of its kind. We propose an interpretable Bayesian inference approach and implement a Poisson model to capture chance occurrences, from which we infer team abilities. We then use a Gaussian mixture model to capture the areas on the pitch a player makes an assist/takes a chance. This approach allows the visualization of differences between players in the way they approach attacking play (making assists/taking chances). We apply the resulting scheme to the 2016/2017 English Premier League, capturing team abilities to create chances, before highlighting key areas where players have most impact.

## Introduction

Within this article, we look to explain an English Premier League team's style of attacking play from data that consist of attacking events only: the details of a chance and the corresponding assist. We determine the number of chances a team creates, along with identifying the players involved and from where on the pitch the chance took place.

The English Premier League is an annual soccer league established in 1992 and is the most watched soccer league in the world.^1,2^ It consists of 20 teams, who, over the course of a season, play every other team twice (both home and away), giving a total of 380 fixtures. It is the top division of English soccer, and every year the bottom three teams are relegated to be replaced by three teams from the next division down (the Championship). In recent times, the Premier League has also become known as the richest league in the world,^3^ through both foreign investment and a lucrative deal for television rights.^4,5^

To compete in the English Premier League, teams employ different styles of play, often determined by the manager's personal preferences and the players who make up the team. Examples of attacking styles of play include counter attacking (quickly moving the ball into scoring range) or passing build-up (many short passes to find a weakness in the opposition's defense). For further discussion of styles of play, we direct the reader to References.^6,7^

Methods to model a soccer team's style of play/behavior have been explored previously by a number of authors. Occupancy maps defined using a given metric (e.g., the mean or an entropy measure of an activity) have been used by Lucey et al.^8^ to determine a team's style of play, with the aim of showing that a team strives to “win home games and draw away ones.” Occupancy maps are also used by Bialkowski et al.,^9^ who take spatiotemporal player tracking data and develop a method to automatically detect formation and player roles.

Player and ball tracking data are used by Lucey et al.^10^ to create spatiotemporal maps of a team's playing style just before they take a shot. Gaussian processes are utilized by Bojinov and Bornn^11^ to form a spatial map to capture each team's defensive strengths and weaknesses. Pass locations are used by Brooks et al.^12^ to determine teams based on their passing styles, before whether pass locations can be used to predict the importance of shots is investigated. All of these methods are applied to dense and expensive data (both in price and in collection expenditure), which consists of all events in a soccer match, with some further utilizing GPS data for the location of the players. Such data are not available to us here.

Methods from the network analysis toolbox are employed by References^13–15^ to draw conclusions about a team/player's use of possession. How the players on a team interact is discussed in Grund^16^ and Kim et al.^17^ estimate the global movements of all players to predict short-term evolutions of play. Outside of soccer, Miller et al.^18^ investigate shot selection among basketball players in the NBA, combining matrix factorization techniques with an intensity surface, modeled using a log-Gaussian Cox process. Defensive play in basketball is captured by Franks et al.,^19^ who take player tracking data and apply spatiotemporal processes, matrix factorization technique,s and hierarchical regression models. Finally, spatiotemporal data are used by Wei et al.^20^ to predict shot locations in tennis.

More generally, the statistical modeling of sports has become a topic of increasing interest in recent times, as more data are collected on the sports we love, coupled with a heightened interest in the outcome of these sports due to the continuous rise of online betting. Soccer is providing an area of rich research, with the ability to capture the goals scored in a match being of particular interest.^21–23^

A player performance rating system (the EA Sports Player Performance Index) was developed by McHale et al.,^24^ which aims to represent a player's worth in a single number, whereas McHale and Szczepański^25^ identify the goal scoring ability of players. Which game-related statistics or performance indicators characterize success (or failure) are determined within References.^26,27^

The length of possession as a predictor of win, lose, or draw was investigated by Jones et al.,^28^ where more successful teams had shorter possessions. Players are rated for a number of abilities, before using them to aid the prediction of goals scored in Whitaker et al.^29^ Finally Kharrat et al.^30^ develop a plus–minus rating system for soccer.

In this article, we propose a method to capture the number of chances a team creates during a given section of a match, along with determining the players involved in a chance, where on the pitch the chance was created and where it was taken from. Our work differs from previous studies in this area in a number of ways. First, previous work has used complete touch data (where every location that a player touches the ball in a game is recorded) to model a team's attacking play. In this study, we use only the location of the assist and the chance. Thus, our proposed method is less computationally intensive and allows inferences from coarser and significantly cheaper data.

Previous work has also focused on modeling the spatial dynamics of a team as a whole, whereas our method identifies the individual spatial contributions of players. Where specific players have been modeled in the past, this is often not accompanied by spatial analysis. Instead, player-to-player relationships are considered. We note that the model proposed within this article has a wide variety of applications, of which we illustrate a few.

The remainder of this article is organized as follows. The data are presented in The Data section. In The Model section, we outline our model to capture a team's chances, before discussing an approach to identify the players involved with each chance and from which spatial locations. Applications are considered in Applications section and a discussion is provided in Discussion section.

## The Data

The data available to us are Stratagem Technologies' Analyst data. This is a collection of data that marks the significant events during a soccer match, including goals, cards (both yellow and red), and chances created. For each of these events, a time is recorded (in minutes), the team and player involved with the event, and for the goals/chances the location on the pitch is marked. If the event is a goal/chance, both the player taking the chance and the player assisting the chance are recorded (along with the spatial location of the chance and the assist). From here on, we consider goals and chances to be the same for our purposes (a goal being a chance that is scored after all)—we refer to them collectively as chance. A section of the data is shown in Table 1. The data cover the 2016/2017 English Premier League season and consist of roughly 32,000 events in total, which equates to ∼85 events for each fixture in the data set. We also have the date of each fixture. We note that the data previously used by References^8,9,11^ to address the types of problems considered in this article typically consist of ∼600,000 events.

**Table 1.** **A section of Stratagem Technologies' analyst data**

*Fixture*	*Date*	*Team*	*Time*	*Type*	*Event player*	*Assist player*	*Assist* x	*Assist* y	*Chance* x	*Chance* y
2241765	August 13, 2016	725	82.35	Yellow card	94,174	—	—	—	—	—
2241765	August 13, 2016	725	81.38	Chance	38,569	38,569	−108	21	−98	34
2241765	August 13, 2016	682	75.65	Chance	5724	11,180	136	41	26	45
2241765	August 13, 2016	682	72.48	Chance	156,662	159,732	47	76	48	39

**FIG. 1.**
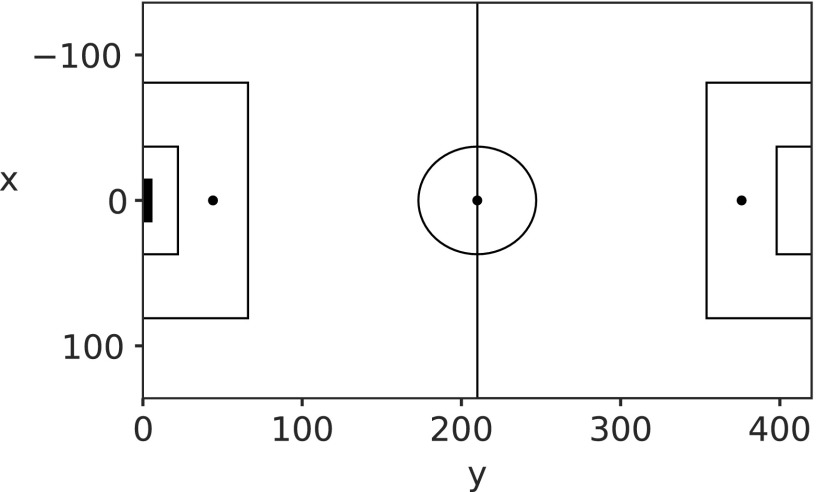
Map of the pitch, the point (0, 0) represents the center of the defended goal (shaded box). Further key reference points are detailed in Table 2.

Locations on the pitch are represented by $( x , y )$ coordinates with the *x*-axis running between the two touch lines (width of the pitch) and the *y*-axis representing the length of the pitch between the goalposts. The spatial location is always recorded from the perspective of the attacking team, meaning the coordinate system does not need to be rotated to account for the second team, or to accommodate the fact that teams switch ends at halftime. The point $( 0 , 0 )$ marks the center of the defended goal, with the width of the pitch running from $- 136$ to 136 (left to right), and the pitch length running from 0 to 420. Explicitly, $x \in [ - 136 , \;136 ]$ and $y \in [ 0 , 420 ]$. A map of the pitch is shown in Figure 1, with some key reference points given in Table 2.

**Table 2.** **Key reference points**

*Point*	x	y
Center of defended goal	0	0
Right goalpost	15	0
Left goalpost	−15	0
6-Yard box, right corner	37	22
6-Yard box, left corner	−37	22
Penalty spot	0	44
18-Yard box, right corner	81	66
18-Yard box, left corner	−81	66
Center spot	0	210

Further to that mentioned, it is possible to extract additional statistics from the data set. These include the game state and the red card state for a team at a given time point. The *game state* is the number of goals a team is winning or losing by at that point in time, for example, a team winning 1-0 would have a game state of +1, a team losing 1–3 would be −2, and, if the game is currently a draw, both teams would have a game state of 0. The *red card state* is defined similarly, and is the difference in the number of players on each team. To elucidate, if a team has a player sent off, its red card state would be −1, whereas the opposition would be +1.

## The Model

In this section, we define our model to capture a team's chances, followed by our approach to determine the composite nature of each individual chance. Each chance consists of an assist player, a player taking the chance (chance player), the spatial location from which the assist was made, and the location of the chance.

**FIG. 2.**
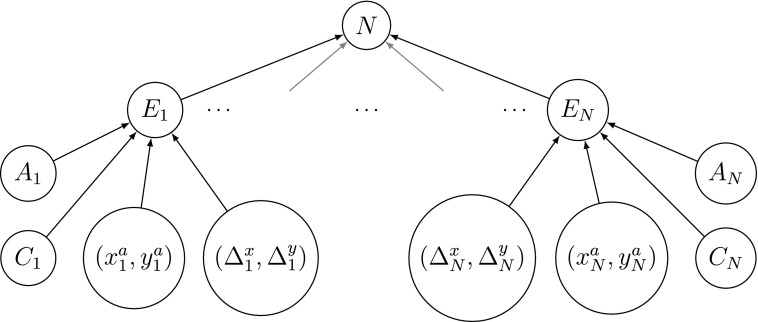
Visual representation of the model for a single team in a given fixture.

First, the number of chances a team has in a given period (*N*) is sampled using a Poisson model. Then for each chance (*E*), we draw an assist player (*A*) and a chance player (*C*) from discrete distributions, with an assist location $( {x^a} , {y^a} )$, and the difference between the assist and chance locations $( { \Delta ^x} , { \Delta ^y} )$ being captured through Gaussian mixture models. A pictorial representation of the components of the model is given in Figure 2. We begin by looking at the number of chances each team generates.

### A team's number of chances

Consider the case wherein we have *K* matches, numbered $k = 1 , \ldots , K$. We denote the set of teams in fixture *k* as ${T_k} \equiv \{  T_k^H , T_k^A \}$, with $T_k^H$ and $T_k^A$ representing the home and away teams, respectively. We take *P* to be the set of all players who feature in the data set, and ${P^j} \in P$ to be the subset of players who play for team *j*.

**FIG. 3.**
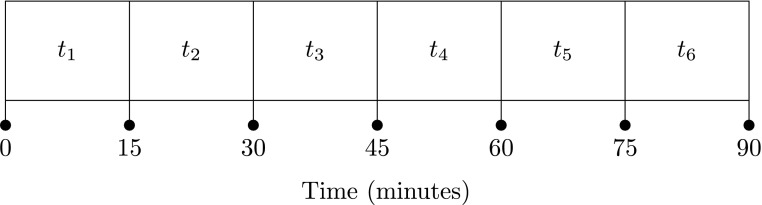
One possible way to split a fixture into blocks.

For simplicity, we outline the model for a single fixture first. We split a fixture into 15-minute blocks, giving six blocks in total (Fig. 3). Our choice of time resolution was decided after a discussion with in-house expert soccer analysts, who agreed that this level of time resolution captures enough temporal variability without unnecessarily complicating the implementation of the model. Typically, a soccer match will have a small amount of extra time at the end of each half. Throughout this article, any chances that occur within these periods of extra time are included in either *t*_3_ or *t*_6_ (using the block structure illustrated in Fig. 3).

For a given match *k*, let $N_{{t_r} , k}^j$ be the number of chances for team $j \in {T_k}$. Let *t_r_*, $r = 1 , \ldots , 6$, denote a particular time block. We have

\begin{align*}
N_{{t_r} , k}^j \;\sim \;{ \rm{Pois}} \left( { \lambda _{{t_r} , k}^j} \right) , \tag{1}
\end{align*}

where

\begin{align*}
\lambda _{{t_r} , k}^j = \exp \left\{  { \theta _{{t_r}}^j - \theta _{{t_r}}^{{T_k} \backslash j} + \left( {{ \delta _{T_k^H , j}}} \right) { \gamma _{{t_r}}} + \alpha G_{{t_r} , k}^j + \beta R_{{t_r} , k}^j} \right\} . \tag{2}
\end{align*}

The notation introduced in Equation (2) is described as follows. A team's baseline propensity to create chances is represented by parameter $\theta _{{t_r}}^j$. Parameter $\theta _{{t_r}}^{{T_k} \backslash j}$ is the opposition's ability to create chances. Parameter ${ \gamma _{{t_r}}}$ is a home effect for the corresponding block and ${ \delta _{a , b}}$ is the Kronecker delta. The home effect reflects the (supposed) advantage the home team has over the away team for the number of goals scored, a phenomenon discussed by numerous authors such as Dixon and Coles^21^ and Karlis and Ntzoufras.^22^ It is not unrealistic to believe that if a home effect exists on the number of goals scored, it will also be prevalent in the number of chances a team creates. The current game state at the start of a block for a team is $G_{{t_r} , k}^j$, with $R_{{t_r} , k}^j$ being the red card state.

For identifiability purposes, we follow Karlis and Ntzoufras^22^ (among others) and impose the constraint that the $\theta _{{t_r}}^j$ must sum-to-zero, over all teams in a given block (*t_r_*). Specifically,

\begin{align*}
\mathop \sum \limits_{i \in j} \theta _{{t_r}}^i = 0.
\end{align*}

The thinking behind this model construction is that if a team is creating chances, the other team cannot. Although this assumption is limiting by construction, given defensive tactics and other tangential aspects of play, it is the easiest (and possibly most meaningful) setup derived from the data, which consists of attacking instances only. From Equations (1) and (2), the likelihood is given by

\begin{align*}
{ L_N } \equiv \prod \limits_ { k = 1 } ^K \prod \limits_ { r = 1 } ^6 \prod \limits_ { j \in { T_k } } { \frac { { { \left( { \lambda _ { { t_r } , k } ^j } \right) } ^ { N_ { { t_r } , k } ^j } } \exp \left( { - \lambda _ { { t_r } , k } ^j } \right) }  { N_ { { t_r } , k } ^j { \kern 1pt } ! } } . \tag { 3 } 
\end{align*}

We note that it is possible to model the number of chances a team creates using an approach similar to that implemented by References^21–23,29^ (although for goals a team scores). However, we find little or no difference in the sum-of-squares, bias, or empirical predictive distributions under the two setups. Thus, we proceed with the simpler model (in terms of the number of parameters) given by Equations (1)–(3).

### Chance composition

Once the number of chances created by a team is determined as mentioned, we break $N_{{t_r} , k}^j$ into separate events, ${E_s} \equiv \left[ {A , C , {x^a} , {y^a} , { \Delta ^x} , { \Delta ^y}} \right]$, where $s = 1 , \ldots , N_{{t_r} , k}^j$, with $E \equiv \left( {{E_1} , \ldots , {E_{N_{{t_r} , k}^j}}} \right) .$ Each *E_s_* is a composition of the assist player (*A*), the chance player (*C*), the $( x , y )$ coordinates for the assist location $( {x^a} , {y^a} )$, and the difference between the assist and chance locations $( { \Delta ^x} , { \Delta ^y} )$, where

\begin{align*}
{ \Delta ^x} = {x^c} - {x^a}
\end{align*}

\begin{align*}
{ \Delta ^y} = {y^c} - {y^a} , \tag{4}
\end{align*}

with $( {x^c} , {y^c} )$ being the $( x , y )$ coordinates of the chance.

First, let us consider the task of determining the assist and chance players involved with each event. We make the assumption that a player cannot assist a player on an opposing team (such as assisting an own goal, by forcing the error), and neither can they take a chance created by a player from the opposition (e.g., running onto a bad back pass). In the context of soccer, these events are reasonably rare, and by implementing this assumption, we can consider the players of one team to be independent from the players of another team (in relation to assisting and taking chances).

A player can switch teams part way through a season (in January) or at the end of a season by means of a transfer. However, we consider them to be a new player to be learned, as they may have different dynamics with their new teammates and possibly play in a different system (e.g., by playing in a new position to that at their previous team). We model the probability of each assist player (and chance player) using a “Multinoulli” (i.e., categorical) distribution.

Let $Z_{s , i , {t_r}}^a$ be a one-hot vector, with a 1 in position *i* representing the assist player for event *s*, in a given block *t_r_*, with $i \in {P^j}$. Denote the probability of each player making an assist for a given event by $\phi _{i , {t_r}}^a$, where

\begin{align*}
\mathop \sum \limits_{i \in {P^j}} \phi _{i , {t_r}}^a = 1.
\end{align*}

Defining $\phi _{{t_r}}^a$ as the vector with entries ${ \{  \phi _{i , {t_r}}^a \}  _{i \in {P^j}}}$, ${ \phi ^a}$ to be the set $\{  \phi _{{t_1}}^a , \ldots , \phi _{{t_6}}^a \}$, $Z_{{t_r}}^a$ as the vector formed from ${ \{  {Z_{1 , i , {t_r}}} , \ldots , {Z_{N_{{t_r} , k}^j , i , {t_r}}} \}  _{i \in {P^j}}}$, and *Z^a^* as the vector defined by $\{  Z_{{t_1}}^a , \ldots , Z_{{t_6}}^a \}$, then

\begin{align*}
Z_{s , i , {t_r}}^a \;\sim \;{\rm Multinoulli} \; \left( { \phi _{{t_r}}^a} \right) , \tag{5}
\end{align*}

with

\begin{align*}
\pi \left( {{Z^a} \vert { \phi ^a}} \right) = \prod \limits_{r = 1}^6 \prod \limits_{s = 1}^{N_{{t_r} , k}^j} \pi \left( {Z_{s , i , {t_r}}^a \vert \phi _{{t_r}}^a} \right) . \tag{6}
\end{align*}

Similarly, for the chance player

\begin{align*}
Z_{s , i , {t_r}}^c \;\sim \;{\rm Multinoulli} \; \left( { \phi _{{t_r}}^c} \right) , \tag{7}
\end{align*}

where

\begin{align*}
\pi \left( {{Z^c} \vert { \phi ^c}} \right) = \prod \limits_{r = 1}^6 \prod \limits_{s = 1}^{N_{{t_r} , k}^j} \pi \left( {Z_{s , i , {t_r}}^c \vert \phi _{{t_r}}^c} \right) . \tag{8}
\end{align*}

Next, we consider the spatial locations, which we model using a mixture model. For a general discussion of mixture models, we refer the reader to McLachlan and Peel.^31^ Given the nature of the spatial locations, we implement a Gaussian mixture model, with *M* components. We denote the weighting of the mixture components (for a given player *i*, in a given block *t_r_*) by $\kappa _{i , {t_r}}^a$ and $\kappa _{i , {t_r}}^ \Delta$ for the assist and $\Delta$ locations, respectively, with $\kappa _{i , {t_r}}^* = ( \kappa _{i , {t_r} , 1}^* , \ldots , \kappa _{i , {t_r} , M}^* )$ and

\begin{align*}
\mathop \sum \limits_{m = 1}^M \kappa _{i , {t_r} , m}^* = 1.
\end{align*}

Furthermore, let the observations for a given player, in a specific block, be $X_{i , {t_r}}^a$ and $X_{i , {t_r}}^ \Delta$, with $X_{i , {t_r}}^* = \left( {X_{i , {t_r} , 1}^* , \ldots , X_{i , {t_r} , L_{i , {t_r}}^*}^*} \right)$. Therefore, the likelihood for the assist locations is

\begin{align*}
{L_a} = \prod \limits_{r = 1}^6 \prod \limits_{i \in P} \prod \limits_{l = 1}^{L_{i , {t_r}}^a} \mathop \sum \limits_{m = 1}^M \kappa _{i , {t_r} , m}^a \times N \left\{   { \left( { \begin{matrix} {x_{i , {t_r} , l}^a} \\ {y_{i , {t_r} , l}^a} \\ \end{matrix} } \right) ; \left( { \begin{matrix} { \mu _{x , m}^a} \\ { \mu _{y , m}^a} \\ \end{matrix} } \right) , \Sigma _m^a} \right\}  , \tag{9}
\end{align*}

where $N ( \cdot { \kern 1pt} ;{ \kern 1pt} m , V )$ denotes the multivariate Gaussian density with mean *m* and variance *V*. Similarly

\begin{align*}
{L_ \Delta } = \prod \limits_{r = 1}^6 \prod \limits_{i \in P} \prod \limits_{l = 1}^{L_{i , {t_r}}^ \Delta } \mathop \sum \limits_{m = 1}^M \kappa _{i , {t_r} , m}^ \Delta \times N \left\{  { \left( { \begin{matrix} {x_{i , {t_r} , l}^ \Delta } \\ {y_{i , {t_r} , l}^ \Delta } \\ \end{matrix} } \right) ; \left( { \begin{matrix} { \mu _{x , m}^ \Delta } \\ { \mu _{y , m}^ \Delta } \\ \end{matrix} } \right) , \Sigma _m^ \Delta } \right\} . \tag{10}
\end{align*}

To simplify our approach, we choose to predetermine the number of components that make up our mixture model. After discussion with inhouse expert soccer analysts, we decided upon eight components, whose locations we determine through k-means clustering. Thus, we set $\mu _m^a$, $m = 1 , \ldots , M$, to be the cluster centroids defined using all the observed assist locations (by all players), and $\mu _m^ \Delta$, $m = 1 , \ldots , M$, using the $\Delta$ locations [deterministically constructed through Equation (4) using the chance and assist locations]. We leave $\Sigma _m^a$, $\Sigma _m^ \Delta$, $m = 1 , \ldots , M$, as parameters to infer, rather than taking the variances of clusters *per se*.

**FIG. 4.**
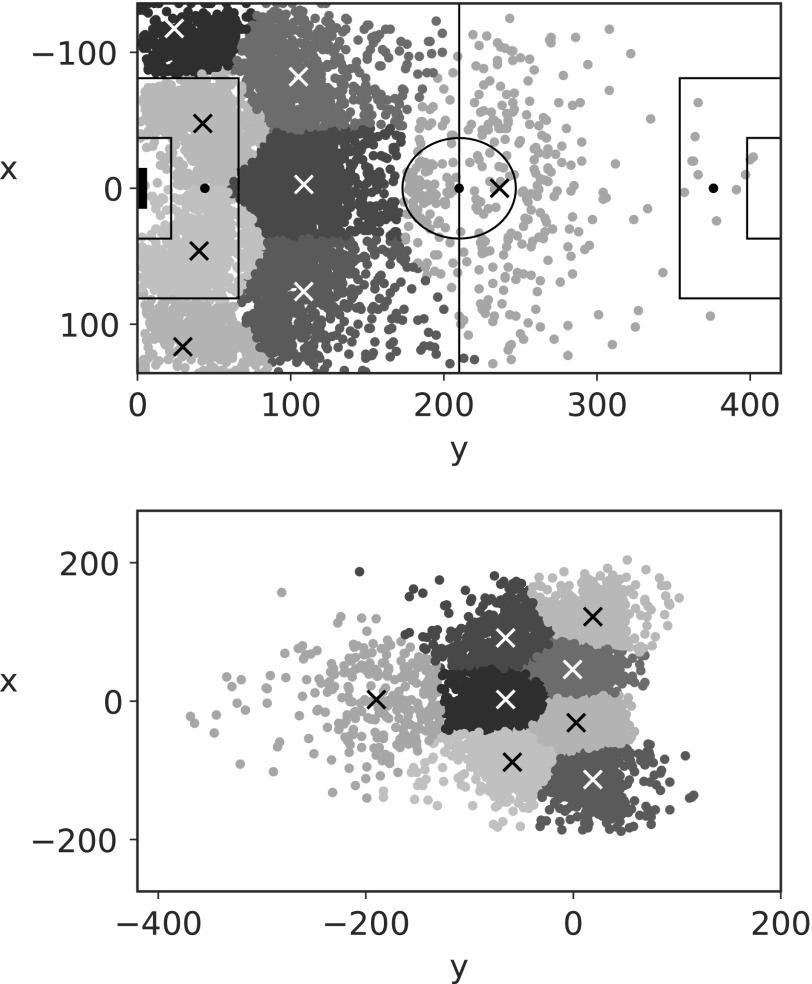
Cluster centroids (cross) under k-means with all data points classified by cluster assignment. Top assist (location of chance creation), bottom $\Delta$ [position of chance in relation to assist location, constructed under Eq. (4)].

The locations of the cluster centroids are shown in Figure 4 (indicated by a cross), where we also plot the data, shaded according to cluster assignment under k-means. We see the different scale on the $\Delta$ plot due to their deterministic construction under Equation (4) (chance location − assist location).

To add some context to the cluster centroids, for the assist locations, the furthest right centroid $( 0 , 240 )$ (own half [OH]) is likely to represent a long ball forward for a player to run on to. For the leftmost column, the widest centroids $( x = -  115 , 115 )$ (left corner [LC], right corner [RC]) are assists from corners or crosses into the box, whereas the middle two (left box [LB], right box [RB]) show cutbacks across goal and knockdowns. The middle column is slightly more ambiguous, although the center cross (center opposition half [CH]) is most likely short through-ball assists, with the wider centroids being free kicks and further crosses into the box (left opposition half [LH], right opposition half [RH]).

The $\Delta$ centroids are the inverse of the assist centroids (in shape) and are simply the distance the ball traveled for the assist, for example, a larger magnitude of *x* and a smaller magnitude of *y* represent a cross into the box. The extremes on the *x*-axis (−200, 200) indicate that the ball traveled across nearly all of the width of the pitch in creating the chance; whereas a *y* value of −400 indicates a long ball forward, for example, from a goalkeeper. A positive value of *y* occurs when a chance is created by passing the ball backwards down the pitch, for instance, a player passing the ball back from the opposition's goal line to the edge of the box.

We note that, although the confines of the pitch are bounded, the mixture of Gaussians has infinite support. Here, we ignore this aspect for computational simplicity and fit flexible covariance matrices to mitigate the effect—through our explorative analysis we believe this effect to be negligible.

Having outlined the two components of our model, namely, the number of chances a team generates and the composition of these chances, we must consider an appropriate way to fit the model, which is the subject of the next section.

### Bayesian inference

To estimate the parameters in the model, we use a Bayesian inference approach. The joint posterior is given by

\begin{align*}
\pi \left( { \theta , \alpha , \beta , \tau , { \phi ^a} , { \phi ^c} , { \kappa ^a} , { \kappa ^ \Delta } , { \Sigma ^a} , { \Sigma ^ \Delta } \vert N , {Z^a} , {Z^c} , {x^a} , {y^a} , { \Delta ^x} , { \Delta ^y}} \right)
\end{align*}

\begin{align*}
\propto \pi \left( \alpha \right) \pi \left( \beta \right) \pi \left( \gamma \right) \pi \left( \tau \right) \pi \left( { \theta \vert \tau } \right) \pi \left( {N \vert \theta , \alpha , \beta , \gamma } \right)
\end{align*}

\begin{align*}
em\times \pi \left( {{ \phi ^a}} \right) \pi \left( {{Z^a} \vert { \phi ^a}} \right) \pi \left( {{ \phi ^c}} \right) \pi \left( {{Z^c} \vert { \phi ^c}} \right)
\end{align*}

\begin{align*}
\times \pi \left( {{ \kappa ^a}} \right) \pi \left( {{ \Sigma ^a}} \right) \pi \left( {{x^a} , {y^a} \vert { \kappa ^a} , { \mu ^a} , { \Sigma ^a} , { \phi ^a}} \right)
\end{align*}

\begin{align*}
\quad \quad \quad \times \pi \left( {{ \kappa ^ \Delta }} \right) \pi \left( {{ \Sigma ^ \Delta }} \right) \pi \left( {{ \Delta ^x} , { \Delta ^y} \vert { \kappa ^ \Delta } , { \mu ^ \Delta } , { \Sigma ^ \Delta } , { \phi ^c}} \right) , \tag{11}
\end{align*}

where $\pi ( N \vert \theta , \alpha , \beta , \gamma )$ follows Equation (3), $\pi ( {Z^a} \vert { \phi ^a} )$ is given by Equation (6) and $\pi ( {Z^c} \vert { \phi ^c} )$ by Equation (8), $\pi ( {x^a} , {y^a} \vert { \kappa ^a} , { \mu ^a} , { \Sigma ^a} , { \phi ^a} )$ is governed by Equation (9) and $\pi ( { \Delta ^x} , { \Delta ^y} \vert { \kappa ^ \Delta } , { \mu ^ \Delta } , { \Sigma ^ \Delta } , { \phi ^c} )$ follows Equation (10). Furthermore, $\pi ( \theta \vert \tau )$ is the prior density ascribed to $\theta$, dependent upon $\tau$, which we take to follow an $N ( 0 , \tau )$ distribution.

To fully specify the model, we implement the following priors

\begin{align*}
\begin{split} & \pi \left( \alpha \right) \;\sim \;N \left( {{{0 , 10}^2}} \right) , \; \; \; \; \; \; \; \; \; \pi \left( \beta \right) \sim N \left( {{{0 , 10}^2}} \right) , \\ & \pi \left( \gamma \right) \;\sim\; N \left( {{{0 , 10}^2}} \right) , \; \; \; \; \; \; \; \; \; \pi \left( \tau \right) \sim {\rm Gamma} ( 1 , 0.01 ) , \\ & \pi \left( {{ \phi ^a}} \right) \;\sim\; {\rm Dirichlet} \left( {{1_P}} \right) , \; \; \; \pi \left( {{ \phi ^c}} \right) \sim {\rm Dirichlet} \left( {{1_P}} \right) , \\ & \pi \left( {{ \kappa ^a}} \right) \;\sim\; {\rm Dirichlet} \left( {{1_M}} \right) , \; \; \pi \left( {{ \kappa ^ \Delta }} \right) \sim {\rm Dirichlet} \left( {{1_M}} \right) , \\ & \pi \left( {{ \Sigma ^a}} \right) \;\sim\; {{ \cal W}^{ - 1}} \left( {{I_2} , 2} \right) , \; \; \; \; \; \; \pi \left( {{ \Sigma ^ \Delta }} \right) \sim {{ \cal W}^{ - 1}} \left( {{I_2} , 2} \right) , \\\end{split}
  \tag{12}
\end{align*}

where ${1_q}$ is a vector of 1s with length *q*, *I_q_* is the identity matrix with dimension *q*, and ${{ \cal W}^{ - 1}}$ is the inverse Wishart distribution. By assuming ${ \phi ^*}$ follows a Dirichlet distribution *a priori*, we are modeling the assist and chance players as a mixture of multinomials within a hierarchical Bayesian model, which is in line with techniques used in topic modeling. Where topic models (usually) capture the words for a particular topic, here, we determine the players for an assist or chance.

The form of Equation (11) admits a Gibbs sampling strategy with blocking, which we can extend to form five independent full conditionals for the number of chances, the assist player, the chance player, the location of the assist, and the $\Delta$ location. Further blocking strategies that exploit the conditional dependencies between the model parameters and the data can also be used. To elucidate, the assist player, ${ \phi ^a}$, can be updated separately for each team. On top of this, all parameters can be updated separately for each block, *t_r_*. We fit the model in Python using the package PyMC3.

## Applications

In the previous sections, we described our model for the spatial composition of goal chances according to latent player abilities. In this section, we test the proposed method in real-world scenarios. Given the independence between the components that constitute the model, we consider two applications. In the first, we learn a team's ability to create chances and, in the second, we examine which players are involved, and where on the pitch these events occur.

For both applications, we use the data described in The Data section, namely the 2016/2017 English Premier League. We note that Chelsea won the league, with Tottenham Hotspur, Manchester City, and Liverpool getting UEFA Champions League places. Therefore, we may expect these four teams to be the best. In contrast, Sunderland, Middlesbrough, and Hull were relegated at the end of the season, meaning these three teams were perhaps the worst.

### Determining a team's chance ability

**FIG. 5.**
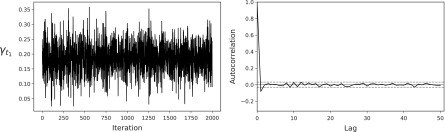
Trace and autocorrelation plots for ${ \gamma _{{t_1}}}$.

We fit the model defined by Equations (1)–(3) using the priors specified in Equation (12). We found little difference in results for alternative priors. We ran the model for 2000 iterations, after an initial burn-in of 100 iterations. Trace and autocorrelation plots for ${ \gamma _{{t_r}}}$ are given in Figure 5, where we observe reasonable mixing (this trace/autocorrelation plot is typical for all parameters in the model).

**FIG. 6.**
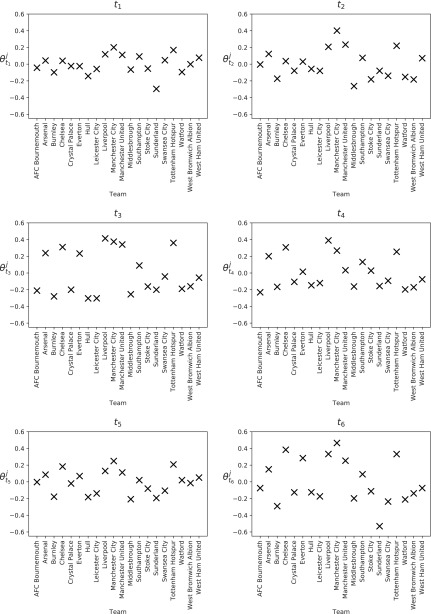
A team's mean ability to create chances, $\theta _{{t_r}}^j$, in the 2016/2017 English Premier League for each block.

The posterior means for a team's ability to create chances ($\theta _{{t_r}}^j$) over the entire 2016/2017 season are presented in Figure 6, with the explicit values given in Table 3, for each of the six blocks. Those teams that we identified as possibly being “better” at creating chances, namely Chelsea, Tottenham Hotspur, Manchester City, and Liverpool, all have higher values in the table. Noticeably, they have higher values for blocks *t*_5_ and *t*_6_, when compared with other teams. This suggests that they are able to find a way to win (by creating more chances) in the closing moments of a game (or a way to recover if they are losing), which is perhaps why they had a successful season.

**Table 3.** **A team's mean ability to create chances, $\theta _{{t_r}}^j$, in the 2016/2017 English Premier League for each block**

	*Block*					
*Team*	t_*1*_	t_*2*_	t_*3*_	t_*4*_	t_*5*_	t_*6*_
AFC Bournemouth	−0.043	−0.004	−0.211	−0.231	−0.003	−0.075
Arsenal	0.043	0.122	0.238	0.201	0.086	0.150
Burnley	−0.098	−0.174	−0.280	−0.166	−0.178	−0.291
Chelsea	0.040	0.036	0.310	0.307	0.183	0.384
Crystal Palace	−0.0231	−0.079	−0.200	−0.106	−0.020	−0.126
Everton	−0.024	0.030	0.233	0.015	0.069	0.284
Hull	−0.143	−0.057	−0.304	−0.147	−0.183	−0.125
Leicester City	−0.058	−0.080	−0.303	−0.121	−0.139	−0.174
Liverpool	0.118	0.207	0.414	0.390	0.130	0.333
Manchester City	0.201	0.401	0.375	0.268	0.249	0.465
Manchester United	0.111	0.234	0.341	0.033	0.112	0.253
Middlesbrough	−0.065	−0.263	−0.255	−0.162	−0.208	−0.198
Southampton	0.092	0.075	0.090	0.132	0.020	0.091
Stoke City	−0.053	−0.182	−0.162	0.028	−0.081	−0.113
Sunderland	−0.296	−0.080	−0.200	−0.156	−0.194	−0.531
Swansea City	0.047	−0.139	−0.042	−0.093	−0.106	−0.236
Tottenham Hotspur	0.169	0.220	0.360	0.254	0.208	0.332
Watford	−0.095	−0.153	−0.189	−0.197	0.020	−0.211
West Bromwich Albion	−0.001	−0.183	−0.160	−0.171	−0.015	−0.138
West Ham United	0.077	0.071	−0.056	−0.076	0.051	−0.075

These are the values shown in Figure 6.

Manchester City has the highest value in *t*_1_, *t*_2_, and *t*_6_, meaning that they started and finished games well. These values highlight Pep Guardiola's playing style, along with the quality of Manchester City's substitutes (they can replace good players with equally good players). Chelsea does not have as high values as some of the other top teams (even though they won the league), suggesting they did not create as many chances as other teams, but they were more clinical with those they did create.

The teams that were relegated at the end of the season (Sunderland, Middlesbrough, and Hull) have some of the lowest values in the table. Sunderland has the worst ability to create chances in *t*_1_ and *t*_6_, with Middlesbrough having a similar ability across all blocks, leading to them being the two lowest scoring teams in the league.

**FIG. 7.**
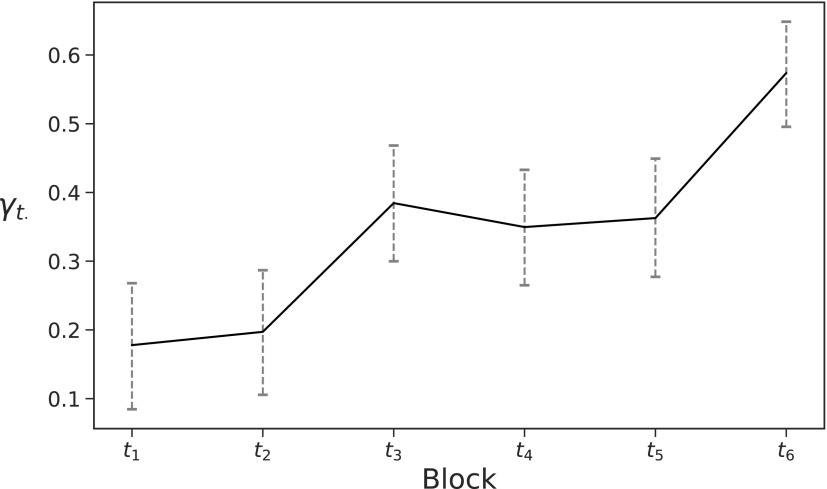
Mean home effect (solid line) and 95% credible intervals (dotted line) in each block in the 2016/2017 English Premier League.

Figure 7 shows the posterior mean for the home effect in each block over the entire 2016/2017 season, along with 95% credible intervals. The credible intervals in each block are of near identical size, meaning that we have similar levels of uncertainty surrounding all ${ \gamma _{{t_r}}}$s. For all blocks, we see a positive home effect, showing a team tends to create more chances at home than when playing away. This is in line with other findings concerning home effects.

There is a rise in the home effect in *t*_3_ (the end of the first half), this is possibly due to fan pressure to perform well. If a team is losing going into half time, fans want to see their team trying to get back into the game (by creating more chances), if they are drawing, they want to try to gain an advantage, or if they are winning, they want to see them press home their advantage. This level of home effect carries into the second half $( {t_4} , {t_5} )$ before a similar rise is observed in *t*_6_. The rise at the end of the game may correspond to a home team's need to achieve a positive result to please their fans. It is also possible that the home team is able to draw more energy from the crowd, and, therefore, outperform the away team toward the end of a match.

The trend seen in Figure 7 complements the findings of Lucey et al.,^8^ which a team will play more defensively away from home, with the suggestion that if an away team is winning or drawing in the final 15 minutes of a game (*t*_6_), they will attempt to hold onto what they have (by defending more and creating less chances).

#### Predicting a team's number of chances

**FIG. 8.**
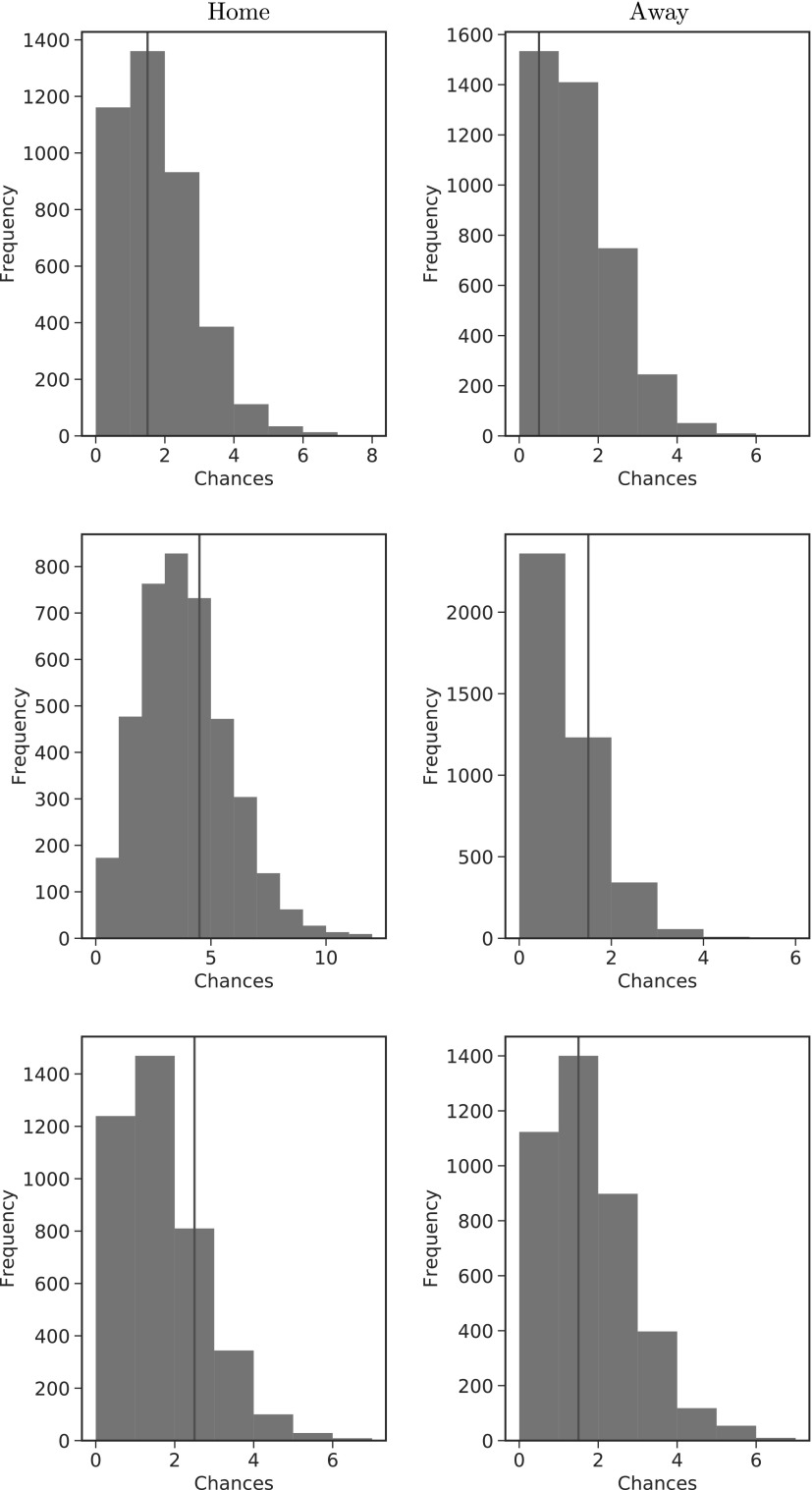
Predictive distributions for the home (left column) and away (right column) teams in three random fixtures and random blocks (each row). The observed value is indicated by the line.

By fitting the model to a certain point in the season and combining parameters, we are able to construct out-of-sample predictive distributions for the number of chances home and away teams create in a given block of a specific fixture. In particular, we fit the model up to a given week and predict the next week's fixtures. The predictive distributions for three randomly selected fixtures and blocks are presented in Figure 8, with the observed value indicated by the line, jittered by adding 0.5 for ease of visualization. From the figure it is clear to see that the observed value falls within the predictive distribution in each case, indicating we have a reasonable model fit and predictive performance.

There is a variety of shape to the distributions, suggesting that the model is able to capture the differences between teams, as well as the differing opposition, and the different blocks in which chances can take place. Overall, the results presented in this section suggest the model is able to capture a team's number of chances reasonably well, as well as offering sensible predictions for the future.

### Determining locations

Having determined the number of chances a team will create, we now fit the model defined through Equations (6) and (8)–(10) to capture the composition of these chances. Initially, we focus our attention on the assist and $\Delta$ locations.

**FIG. 9.**
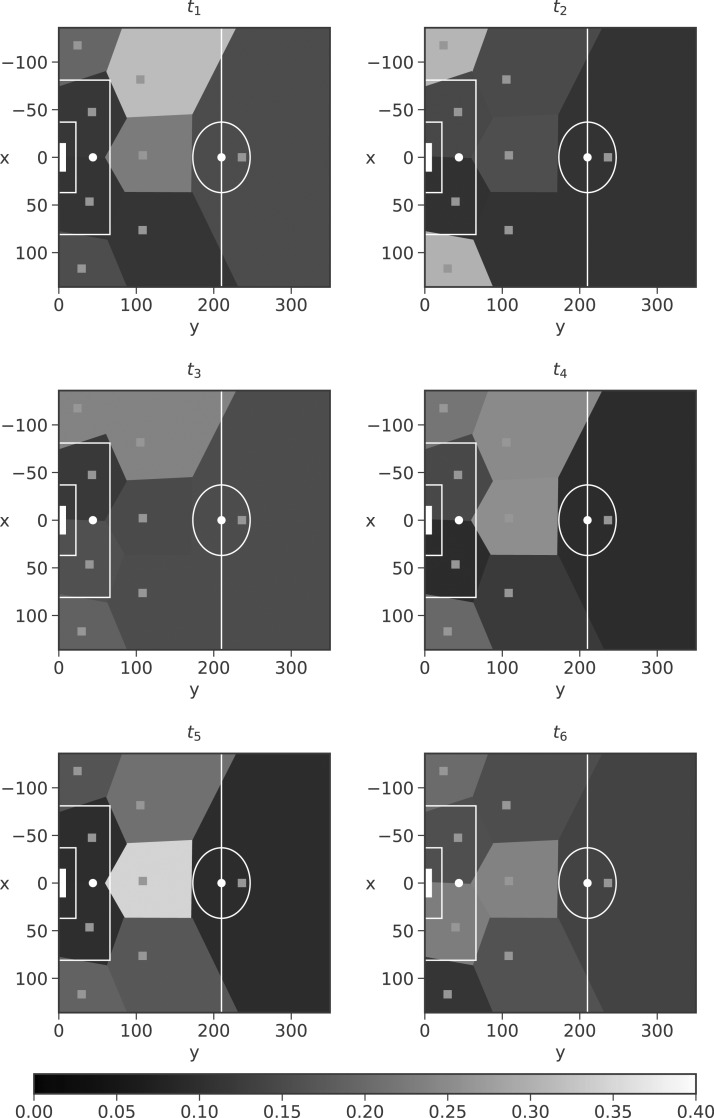
Eriksen assist locations for each block in the 2016/2017 English Premier League, shaded according to the mean weighting of each mixture component.

Christian Eriksen created the most chances in the 2016/2017 English Premier League. Figure 9 shows the locations of these assists in each block through a Voronoi diagram, shaded according to the mean weighting of each mixture component $\big( { \kappa _{i , {t_r}}^a}\big )$. There is evidence that Eriksen changes his style of play (or at least the location of his play, and possibly effectiveness) during different periods of the game, for example, the plots for *t*_1_ and *t*_2_.

As we are implementing the model within the Bayesian paradigm, we can fit the model to a certain point in the season, before updating our beliefs once more data become available (more matches are played). To this end, we learn the model parameters using data up until January 1, 2017 (roughly half the season), and then proceed to update our beliefs after each subsequent month.

**FIG. 10.**
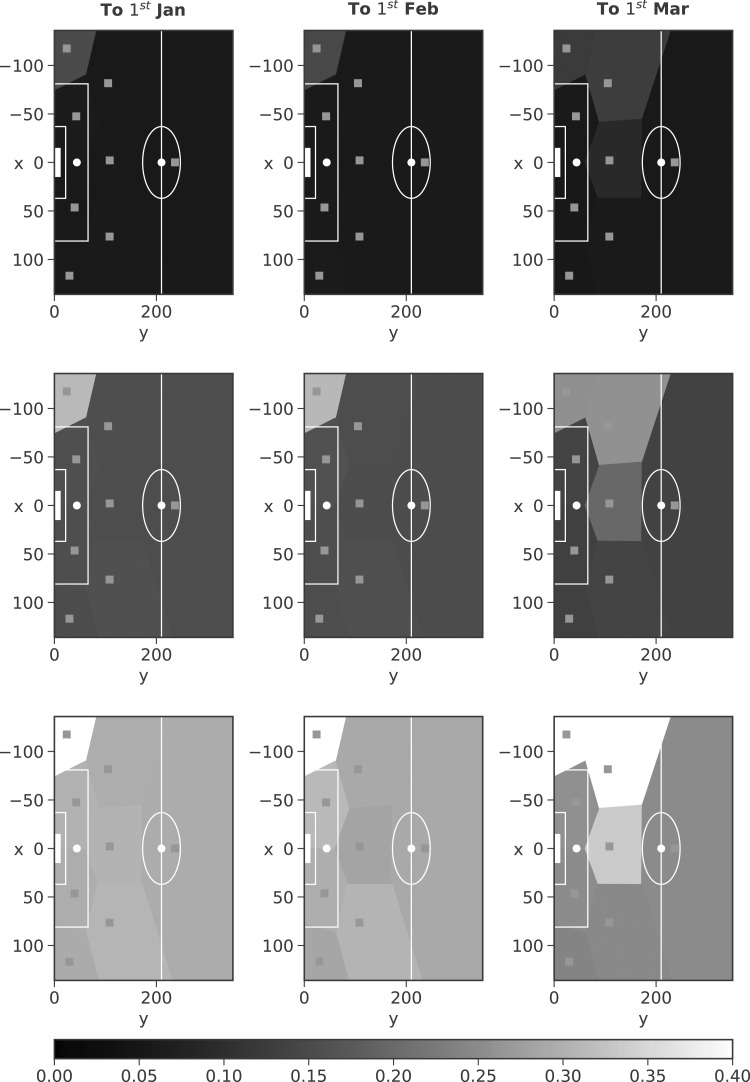
Mahrez assist locations in *t*_5_ after different periods of time, shaded according to the weighting of each mixture component. Top row weights based on 5% quantile, middle row weights based on the mean, and bottom row weights based on 95% quantile.

**FIG. 11.**
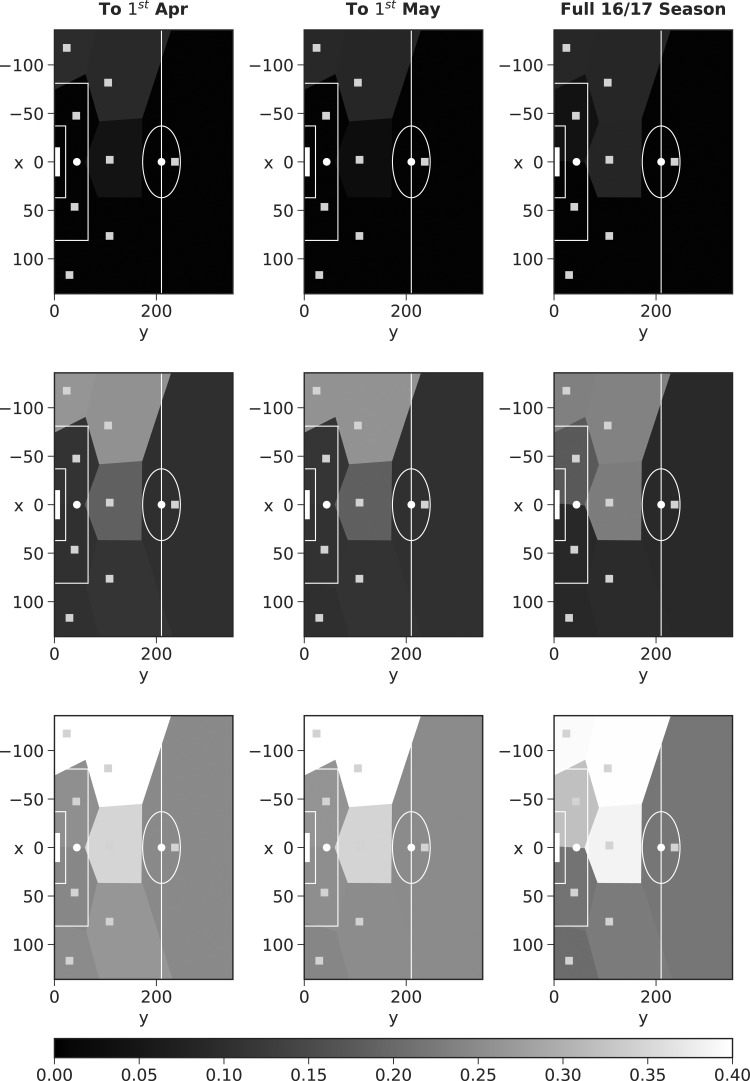
Mahrez assist locations in *t*_5_ after different periods of time, shaded according to the weighting of each mixture component. Top row weights based on 5% quantile, middle row weights based on the mean, and bottom row weights based on 95% quantile.

Voronoi diagrams for Riyad Mahrez's assists in *t*_5_ after each of these months (along with the season as a whole) are shown in Figures 10 and 11. Mahrez was one of the stars for Leicester City when they won the league in 2015/2016; however, he was not playing as well under manager Claudio Ranieri in 2016/2017 (our data set); this is suggested by the middle row of plots shown in Figure 10 (based on the mean weights), where high weights are only assigned to the LC. Ranieri was sacked in February and Craig Shakespeare became manager, who was seen to get Mahrez back to playing somewhere near his best. The figures support this, with the middle row of Figure 11 (again based on the mean weights) showing assists coming from more areas of the pitch, those being, the left-hand side, drifting to more central positions.

Utilizing the posterior densities, we can assess the uncertainty surrounding the weighting of each mixture component. The top row of Figures 10 and 11 shows Voronoi diagrams based on the 5% quantile of the marginal posterior densities for the weights, whereas the bottom row is formed using the 95% quantile. We see that as the season progresses, from the first fit (to January 1) to the full 2016/2017 season fit, the distance between the plots constructed under the 5% and 95% quantiles narrows (the plots look more similar). This is an advantage of implementing the model within the Bayesian paradigm; we are able to update our beliefs as more data become available, and reduce uncertainty.

We note that we observe only a small reduction in the variance here, however, this is due to the small number of observations available for each player. Fitting the model through time, over multiple seasons, would help to reduce the uncertainty surrounding individual player's mixture weights (subject to the data being available). This approach (through Figs. 9–11) illustrates that we can model how a player plays throughout a game and over a season. Given we can update as more data become available, this allows us to capture when players change their style of play or when they start to become more/less important to a team.

**FIG. 12.**
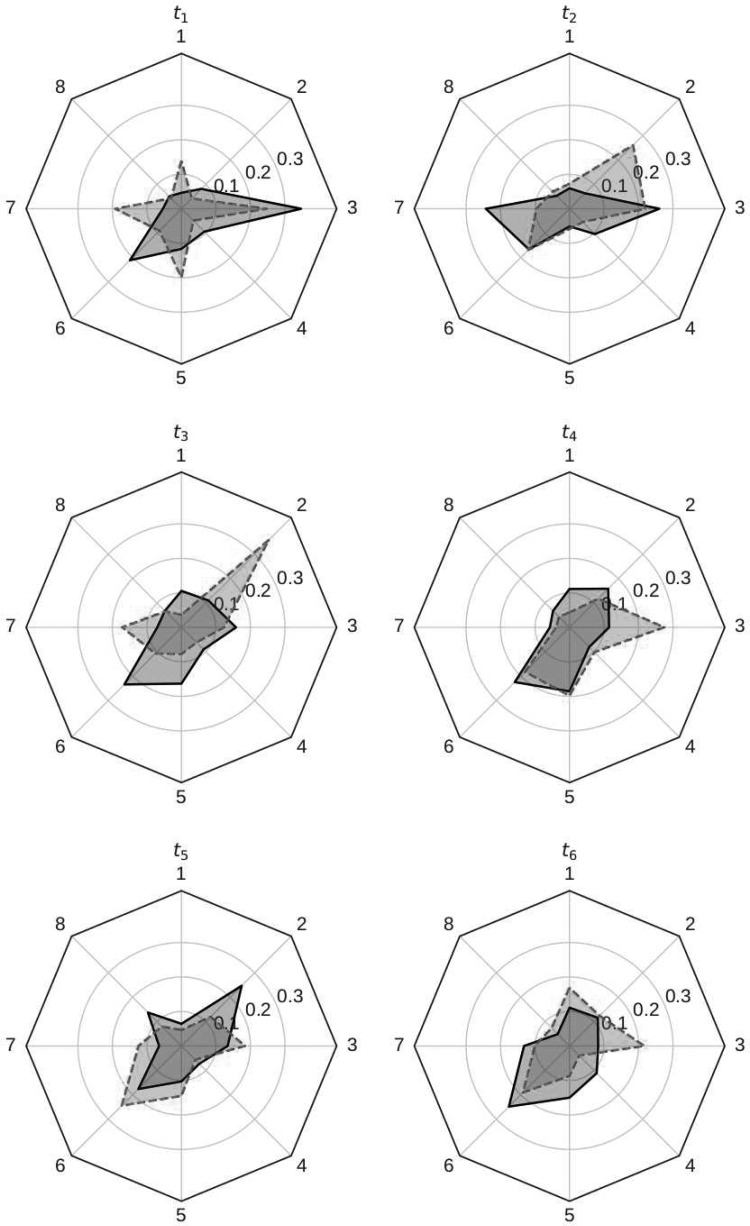
Radar plots of the mean $\kappa _{i , {t_r}}^ \Delta$ for Kane (solid) and Agüero (dashed) for each block in the 2016/2017 English Premier League.

Integrating over the posterior uncertainty of the spatial locations gives the marginal posterior densities for ${ \kappa ^ \Delta }$, from which we can ascertain differences in how certain players take chances. Radar plots of the mean $\kappa _{i , {t_r}}^ \Delta$ (at each centroid) for Harry Kane (scored the most goals) and Sergio Agüero (had the most chances) are shown in Figure 12. For simplicity we number the centroids 1–8. The meaning of each centroid is subtle, and explanation is beyond the scope of this article. However, it is clear from Figure 12 that it is easy to visualize (and distinguish) between how certain players take chances, for instance, the differences in the shape of each player's radar plot.

**FIG. 13.**
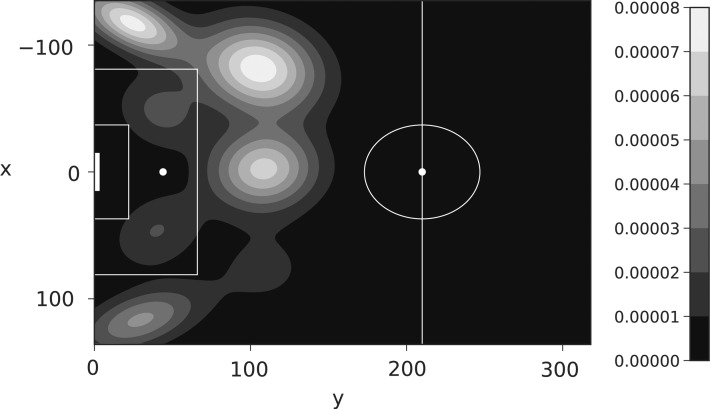
Eriksen assist locations under the Gaussian mixture model for *t*_1_ in the 2016/2017 English Premier League.

By marginalizing over the mixture weights $\left( {{ \kappa ^*}} \right)$, along with the uncertainty within the mixture components, we can construct a surface under the Gaussian mixture model for each player. One such surface is presented in Figure 13. This is the surface for Christian Eriksen's assists in *t*_1_ over the entire 2016/2017 English Premier League. Note, these surfaces can be constructed at any point in the season and updated once more data become available. From the figure, it is easy to see where this player had most influence, and we observe a pattern similar to that seen in the top left plot of Figure 9. Such plots are a useful way to convey information to a team, an application of which we consider hereunder.

#### Identifying a team's strengths and weaknesses

During the 2016/2017 English Premier League, many pundits questioned the ability of Liverpool's defense, highlighting a weakness on the left-hand side. Looking at the data, this criticism appears fair. Of the goals Liverpool concede, the assist leading to the goal is most likely to come from the left-hand side of the box (LB), with a $\Delta$ $( x , y )$ location of approximately (50, 0), see Figure 4 for cluster locations. Moreover, they are most likely to concede from these positions in blocks *t*_3_ and *t*_5_. Therefore, when approaching a game, Liverpool may want to know which of the opposition players are most likely to be involved in chances at these locations for each block, so that they can attempt to reduce their impact.

Let us consider the match Liverpool versus Crystal Palace (April 23, 2017)—Crystal Palace is a team that in recent years has caused Liverpool problems. We fit our model using all data available before the match is played. From the model, in both *t*_3_ and *t*_5_, we expect Crystal Palace to have one chance against Liverpool (in the match they had two chances in both *t*_3_ and *t*_5_).

By integrating over ${ \phi ^*}$, ${ \kappa ^*}$, and ${ \Sigma ^*}$, and by applying Bayes theorem, we can calculate the probability of each player being involved in a chance, for each block, at Liverpool's weak locations. Christian Benteke is the most likely Crystal Palace player in *t*_3_ to have a chance at the $\Delta$ location (with probability 0.166). Andros Townsend is the most likely player in *t*_5_, although there is little difference between the probability of Townsend and Benteke. Assists are likely to come from James McArthur in *t*_3_, or Yohan Cabaye or Jason Puncheon in *t*_5_ (with probabilities 0.134 and 0.121, respectively).

**FIG. 14.**
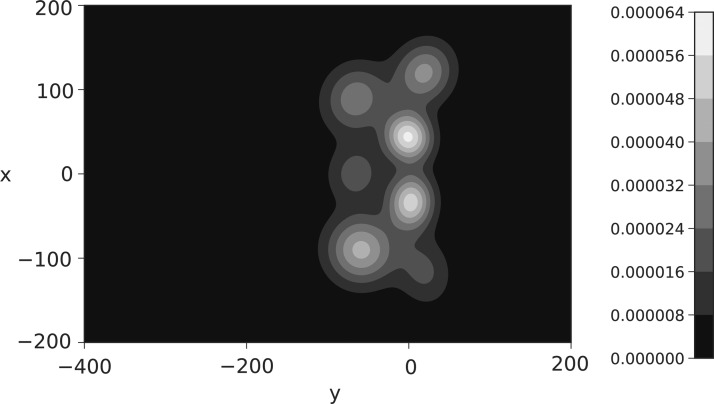
Benteke $\Delta$ locations under the Gaussian mixture model in *t*_3_ using data to April 22, 2017.

**FIG. 15.**
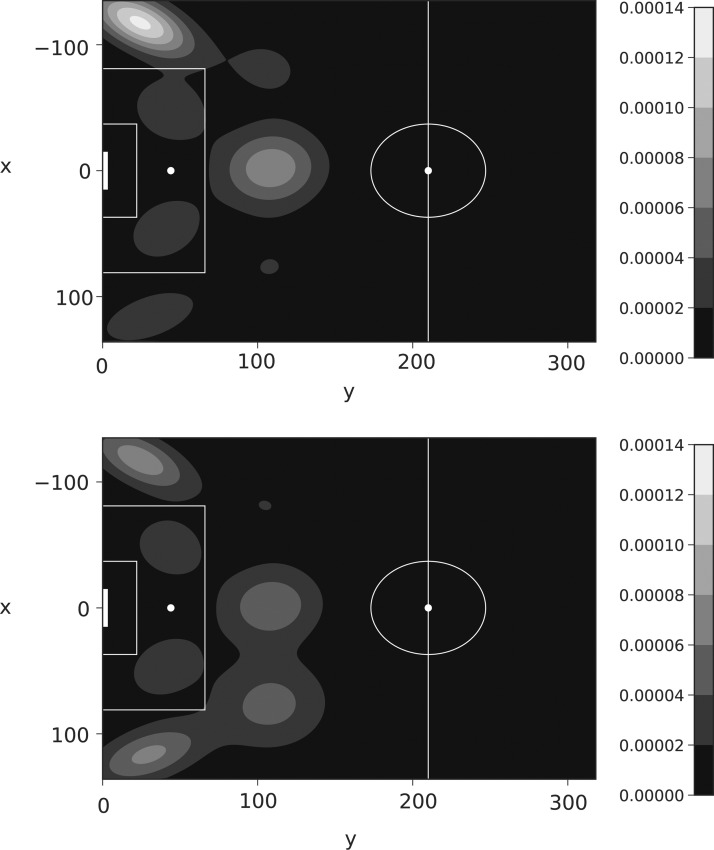
Assist locations under the Gaussian mixture model in *t*_5_ using data to April 22, 2017. Top Cabaye, bottom Puncheon.

The $\Delta$ surface for Benteke in *t*_3_ is shown in Figure 14, with the assist surfaces for Cabaye and Puncheon in *t*_5_ given in Figure 15. In both figures, we see the highlighted ability of these players at the locations Liverpool is most susceptible. During the game, Liverpool did not stop these players adequately enough, with Benteke scoring in both *t*_3_ and *t*_5_, Cabaye assisting in *t*_3_, and Puncheon assisting in *t*_5_.

#### Considering a team as a whole

In a similar style to Lucey et al.^8^ and Bojinov and Bornn,^11^ we can create maps to represent how a team plays overall. Our maps differ from those of References^8,11^ as they use complete touch data, whereas in this study we just use the locations of the chance and the assist. Our maps are, however, conceptually similar.

Integrating over ${ \phi ^a}$, ${ \kappa ^a}$, and ${ \Sigma ^a}$, calculating the probability of each player making an assist, and multiplying the weighting of each mixture component by the requisite probability allow us to create a surface for the assist locations of a team as a whole.

**FIG. 16.**
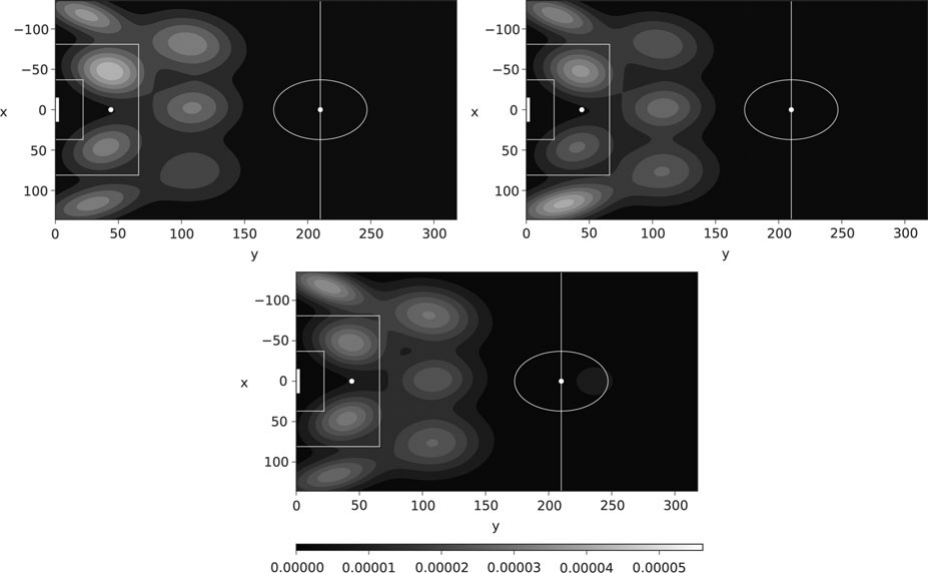
Assist location maps for three teams using data until March 1. Top left Chelsea, top right Manchester City, bottom Burnley.

Figure 16 shows three such surfaces for Chelsea, Manchester City, and Burnley (using data until March 1). It is clear from the figure that this approach allows us to distinguish differences in the way teams play. Burnley is more regimented (and static) than either Chelsea or Manchester City; they make assists from clearly defined locations, and even create chances from long balls forward (the lighter circle in their own half).

Chelsea and Manchester City are more free flowing in their assist making, and create more chances than Burnley. They have better players who can create chances all over the pitch, and as they are known for a passing style, they rarely rely on a long ball forward. Chelsea appears to favor the left box position for assists, whereas Manchester City uses the right corner and left box. The contours of both Chelsea and Manchester City are less tightly ringed than those of Burnley. We believe that these plots show that it is possible to make inferences about the way in which teams attack, distinguishing between teams, in a way similar to References,^8,11^ but from much coarser data.

## Discussion

In this article, we provided a framework to infer how goal chances are created by a team, characterized by spatiotemporal player-level behavior on assisting a play or receiving assists. All is done within a Bayesian inference setting that by construction provides uncertainty measures and the ability to incorporate prior knowledge. We illustrated its value to professional analysts by showing how it can be used to visualize team behavior.

We envisage that such modeling and inference methods can be easily incorporated into the toolbox of any organization with an interest in soccer analytics, due to its simplicity of implementation that can be accomplished by off-the-shelf tools such as the Python package PyMC3. Our approach is computationally efficient and utilizes the combination of a Poisson and Gaussian mixture model.

We have shown in Applications section that inferences under the model are reasonably accurate and have close ties to reality, along with implementable applications, of which we only illustrate a few. In contrast to previous work, we exploit coarser data (consisting solely of attacking events) to identify individual player contributions, rather than modeling the spatial dynamics of a team as a whole.

There are a number of ways in which this work can be extended. First, smoothing techniques can be applied to ${ \phi ^*}$ and ${ \kappa ^*}$ so that the probabilities of players and mixture components vary smoothly over time. This was not implemented here for computational simplicity. We note that, for computational ease, we chose to fix the means of the mixture components. In the future, this simplification could be relaxed and the means inferred simultaneously with the other parameters; the cluster centroids from observed data could be used, for instance, as initial values in the Gibbs sampler.

There is some dependence between the player assisting the chance and the player taking the chance. That is, it is expected that some players link up better with some players than others, further determined by the areas on the pitch in which they play. This dependence between *A* and *C* needs incorporating into the model, which could also allow some network analysis techniques to be implemented. Finally, as an extension to the applications for the proposed method, an interesting area of future work is anomaly detection. This would allow us to highlight a player's patterns that are unusual in given fixtures and blocks. Techniques discussed in Heard et al.^32^ could be used as inspiration for methods to detect these changes.

## Abbreviations Used

CH: center opposition half
LB: left box
LC: left corner
LH: left opposition half
OH: own half
RB: right box
RC: right corner
RH: right opposition half
